# A Fluorescent Coumarin-Based Probe for the Fast Detection of Cysteine with Live Cell Application

**DOI:** 10.3390/molecules22101618

**Published:** 2017-09-26

**Authors:** Rui-Feng Zeng, Jin-Shuai Lan, Xiao-Die Li, Hui-Fen Liang, Yan Liao, Ying-Jie Lu, Tong Zhang, Yue Ding

**Affiliations:** 1 School of Pharmacy, Shanghai University of Traditional Chinese Medicine, Shanghai 201203, China; ZRF0049@163.com (R.-F.Z.); 18017984650@163.com (X.-D.L.); nuoanuo@126.com (H.-F.L.); 15216669831@163.com (Y.L.); fgbddxc@126.com (Y.-J.L.); 2 Experiment Center of Teaching & Learning, Shanghai University of Traditional Chinese Medicine, Shanghai 201203, China; lanjinshuai_shut@126.com

**Keywords:** cysteine, fluorescent probe, coumarin, bioimaging

## Abstract

A new coumarin-based fluorescent probe, containing an allylic esters group, has been designed and synthesized for sensing cysteine in physiological pH. In this fluorescent probe, the coumarin was applied as the fluorophore and an allylic esters group was combined as both a fluorescence quencher and a recognition unit. The probe can selectively and sensitively detect cysteine (Cys) over homocysteine, glutathione, and other amino acids, and has a rapid response time of 30 min and a low detection limit of 47.7 nM. In addition, the probe could be applied for cell imaging with low cytotoxicity.

## 1. Introduction

Biothiols, such as cysteine (Cys), homocysteine (Hcy), and glutathione (GSH), are natural compounds containing sulfydryl, which play crucial roles in the process of intracellular protein turnover [1]. Generally, alternations of the level of cellular biothiols lead to many diseases such as psoriasis, leucocyte loss, cardiovascular disease [2], acquired immunodeficiency syndrome (AIDS) [3], psoriasis-like skin lesions [4], lethargy, liver damage [5], muscle weakness, and wasting [6]. In addition, GSH is the vastest non-protein thiol in cells [7], serving as an important regulator of the redox reaction in vivo and maintaining intracellular redox activities [8,9], which is a key indicator of diseases such as diabetes and Alzheimer’s disease [10]. The level of Hcy has been proved to be connected with neural tube defects, Alzheimer’s disease, osteoporosis, and inflammatory bowel disease [11]. What is more, Cys is a semi-essential amino acid that plays a structural role in many proteins, and its concentration is closely linked with edema, slowed growth [12], and a wide variety of cancers including breast cancer [13], colorectal cancer [14], nasopharyngeal cancer [15], and lung cancer [16]. Therefore, it is essential to develop a reliable method for detecting biothiols [17].

Numerous assays have been carried out to detect biothiols including high performance liquid chromatography [18,19], mass spectrometry [20,21], colorimetric detection [22], electrochemical method [23,24], and capillary electrophoresis [25]. However, these studies faced a number of difficulties such as high facility cost, complicated operation, complex preparation, long detecting time, or poor selectivity. Recently, more attention had been paid to developing fluorescent probes, a cheap and easy method to distinguish biothiols [26,27,28]. For example, Tong et al. synthesized a visible blue-to-green pyrene probe, which could detect polyanion in aqueous media but cannot distinguish Cys from other biothiols [29]. Zhao et al. designed a rapid and sensitive probe for Cys, GSH, and Hcy, but it could not identify them from each other [30]. Only a few reports detected Cys from GSH and Hcy due to their similar structures containing nucleophilic mercapto groups [31,32]; thus, a new kind of fluorescent probe with steady, high selectivity and sensitivity is still needed.

Coumarin is a compound containing the parent nucleus of benzopyrone, an economic chromophore. What is more, coumarin shows high intense fluorescence, good solubility, relatively high fluorescence quantum yield, ease of production, and molar absorption coefficient if an electron donating group, such as hydroxy or unalkylated amino group, is substituted at the 7 position [33]. The fluorescence intensity of coumarins has a close relationship with the substituent groups on the ring. When the intramolecular charge transfer capability was changed by modifying substituent groups, the optical properties of the whole molecule were influenced. So, we can control the optical properties of molecules by adding different substituent groups with pushing or pulling electronics. Moreover, various mechanisms have been used in the design of Cys fluorescent probes, including the cyclization reaction with an aldehyde [34,35,36], Michael addition [37,38,39], and cleavage reaction [40,41,42] by thiols.

In this report, a turn-on fluorescence probe based on coumarins was designed for Cys with highly selectivity and sensitivity. The allylic esters group serves as a blocker of coumarin fluorophore in the probe, which not only functions as a Michael receptor, but also an electrophile [43]. Because Cys has lower steric hindrance to conduct Michael additions, the probe can sharply distinguish Cys from other biothiols within 30 min and has been successfully used in living cell imaging. With the development of fluorescent probes for the discrimination of Cys from other amino acids and biothiols, real-time monitoring of the Cys level of cells, tissues, and animals will be possible.

## 2. Results and Discussion

### 2.1. Characterization of the Probe

**Scheme 1:**
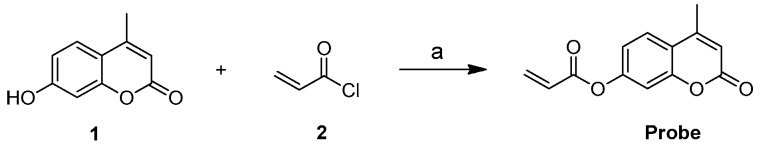
Synthesis of the probe. Reagents and conditions: (a) trimethylamine, CH_2_Cl_2_, r.t.

The synthesis of the probe is outlined in Scheme 1. Compound **1** was further treated with acryloyl chloride to form the probe. The structure of the probe was confirmed by ^1^H-NMR, ^13^C-NMR, IR, and MS spectra (Figures S1–S4). The probe was colorless and had two absorption bands centered at 278 nm and 312 nm. It showed weak fluorescence (Φ = 0.0194) due to the quencher acrylamide group.

### 2.2. UV-Vis Absorption and Fluorescence Spectra

**Figure 1:**
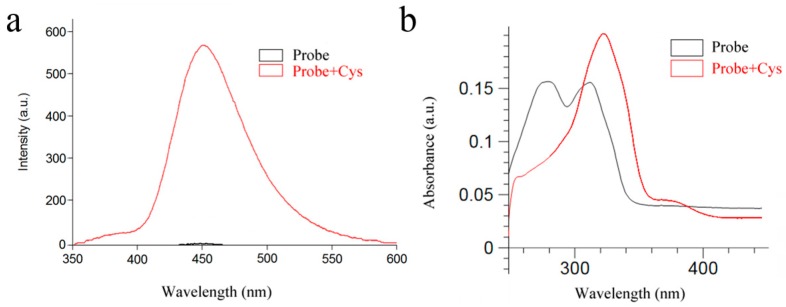
(**a**) The fluorescence intensity of the probe and the probe added to Cys-buffered solution (PBS (phosphate buffered solution):DMSO (dimethyl sulfoxide) = 6:4, pH = 7.4) at room temperature; (**b**) The absorption spectra of the probe and the probe added to Cys in buffered solution (PBS:DMSO = 6:4, pH = 7.4) at room temperature. λ_ex_ = 325 nm.

As shown in Figure 1b and Figure S5, the UV-vis absorption spectra of the free probe (10 μM) showed absorption peaks at 278 nm and 312 nm. Upon the addition of Cys, the absorption at 278 nm gradually decreased, while the absorption at 312 nm increased and red-shifted to 323 nm. Furthermore, as shown in Figure 1a, the fluorescence intensity of the probe was enhanced from Φ = 0.0194 to Φ = 0.1725 with Cys.

### 2.3. Selectivity of the Probe for Cys

**Figure 2:**
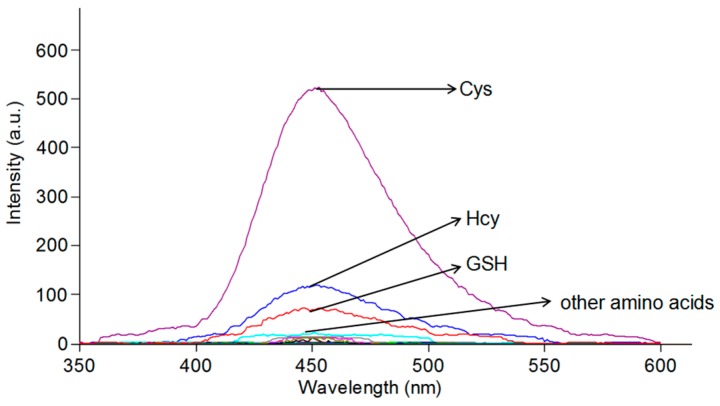
Fluorescence intensity of the probe (10 μM) in the presence of different compounds (50 equiv.); each reaction was in buffered solution (PBS:DMSO = 6:4, pH = 7.4) at room temperature. λ_ex_ = 325 nm.

The sensing ability of the probe towards various compounds was detected, including cysteine (Cys), homocysteine (Hcy), glutathione (GSH), glycine (Gly), lysine (Lys), histidine (His), alanine (Ala), tyrosine (Try), cystine (Cys C), valine (Val), isoleucine (Ile), glutamic (Glu), phenylalanine (Phe), serine (Ser), threonine (Thr), aspartic (Asp), methionine (Met), proline (Pro), leucine (Leu), CN^−^, SCN^−^, HS^−^, and SO_4_^2−^ in buffered solution (PBS (phosphate buffered solution):DMSO (dimethyl sulfoxide) = 6:4, pH = 7.4). In Figure 2, the green fluorescence response was sensed upon the addition of Cys (50 equiv.) to the probe (10 μM), and no obvious changes in fluorescence emission were observed in other compounds. The results indicate that Cys is more active in the reaction due to its stronger nucleophilicity compared with that of GSH and Hcy.

**Figure 3:**
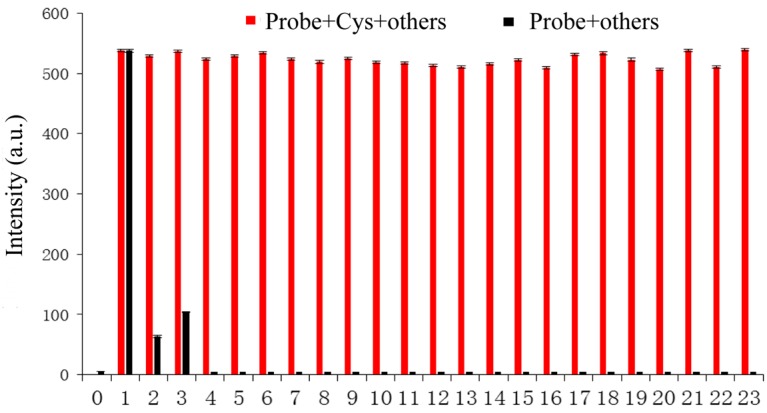
Black bar represents the fluorescence response of the probe (10 μM) to various compounds (50 equiv.). Red bar represents the fluorescence response of the probe (10 μM) to Cys (50 equiv.) in the presence of other compounds (50 equiv.). Each reaction was in buffered solution (PBS:DMSO = 6:4, pH = 7.4) at room temperature. The numbers represent analytes: 0. Blank; 1. Cys; 2. Hcy; 3. GSH; 4. Asp; 5. Val; 6. Glu; 7. Pro; 8. Gly; 9. Phe; 10. Met; 11. Thr; 12. Ser; 13. Ile; 14. His; 15. Phe; 16. Lys; 17. Try; 18. Leu; 19. Cys C; 20. CN^−^; 21. SCN^−^; 22. HS^−^; 23. SO_4_^2−^. λ_ex_ = 325 nm, λ_em_ = 450 nm.

In order to ensure an accurate detection of Cys, the probe could not be obstructed by other compounds. Cys (50 equiv.) and a series of competing compounds (50 equiv.) were added to the probe (10 μM). As depicted in Figure 3, other compounds hardly had an influence on the fluorescence emission. The results showed that the probe could sharply distinguish Cys from the other compounds.

### 2.4. Effect of pH on the Fluorescence Response of Probe

**Figure 4:**
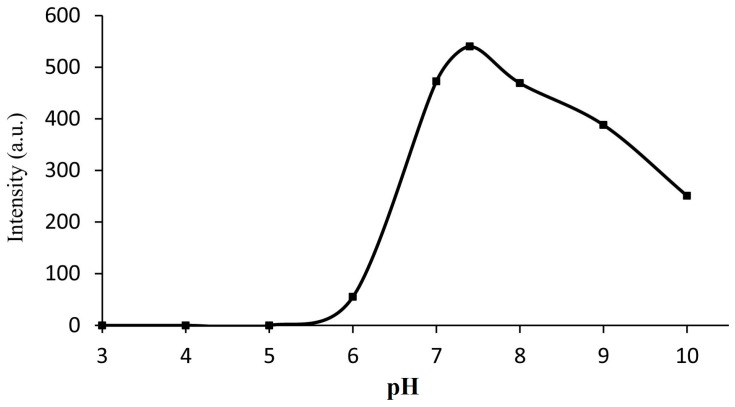
The effect of pH on the fluorescence response of the probe (10 μM) in the presence of Cys (500 μM) in buffered solution (PBS:DMSO = 6:4, pH = 3–10), pH was adjusted by NaOH and HCl. λ_ex_ = 325 nm, λ_em_ = 450 nm.

To confirm the appropriate pH scope of the probe, the fluorescence intensity of the probe (10 μM) added to Cys (50 equiv.) with different values of pH (3–10) was detected (Figure 4). The result showed that the range of pH 7–9 fitted the reaction well. Because pH 7.4 is close to physiological conditions, we selected a buffered solution at this level (PBS:DMSO = 6:4, pH = 7.4) for subsequent study.

### 2.5. Effect of Reaction Time

**Figure 5:**
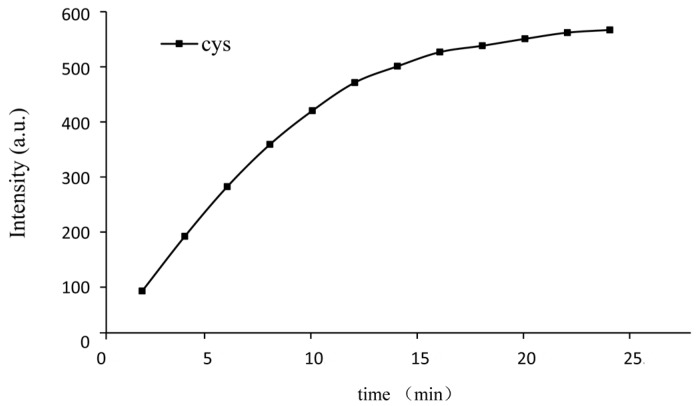
Kinetic analysis of the probe towards Cys (10 μM probe with 50 equiv. of Cys) in buffer solution (DMSO:PBS = 4:6, pH = 7.4). λ_ex_ = 325 nm, λ_em_ = 450 nm.

To track the proper time of the response, we traced the fluorescence intensity of the probe (10 μM) added to Cys (50 equiv.) while monitoring the time. The fluorescence intensity almost reached the maximum in 15 min (Figure 5), which was relatively fast. Furthermore, the response obeyed the pseudo-first-order rate, and the rate constant was calculated according to the following equation [44]:

Ln ((F_max_ − F) / F_max_) = −kt, (1)

where F is the fluorescence intensity at time t, and F_max_ is the fluorescence intensity after the reaction totally completed. The constant k is shown in Figure S6. The value of the rate constant of Cys was 0.1618 min^−1^.

### 2.6. Quantitative Responses of the Probe for Cys

**Figure 6:**
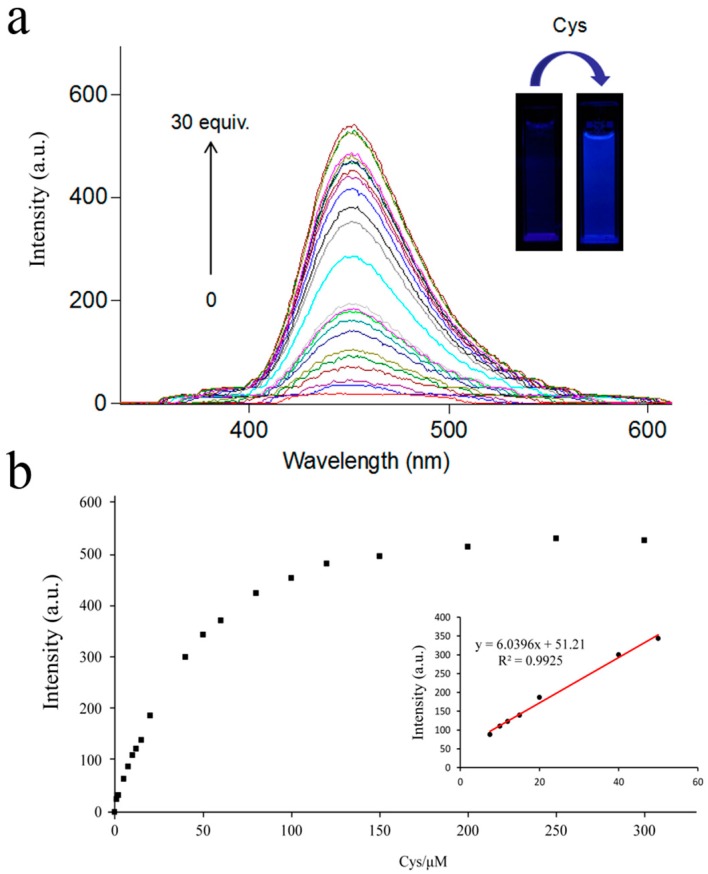
(**a**) Fluorescent spectral changes (**b**) The fluorescence intensity at 450 nm of probe (10 μM) upon addition of Cys (0–300 μM) in buffer solution (DMSO:PBS = 4:6, PH 7.4), incubated 30 min before detected. λ_ex_ = 325 nm, λ_em_ = 450 nm.

To clarify the sensitivity of the probe, various concentrations of Cys (0–30 equiv.) were added to the probe solution (10 μM) and the fluorescence intensity at 450 nm was recorded after 50 min. The results changed from nearly dark to a strong green fluorescent response as the concentration of Cys increased. Furthermore, the emission intensity at 450 nm was linearly related to the concentration of Cys from 0 to 50 μM (R^2^ = 0.9925) (Figure 6), which provided useful conditions for the quantitative analysis of Cys. And the detection limit of Cys was calculated to be 47.7 nM based on the 3σ/slope method [45]. The results indicated that the probe remained very sensitive to Cys.

As described above, the probe displayed excellent analytical properties, compared with some other fluorescent probes of recent reports, for the detection of Cys. The comparison data is listed in Table 1, indicating that the probe is promising for practical analysis.

### 2.7. Reaction Mechanism

**Scheme 2:**
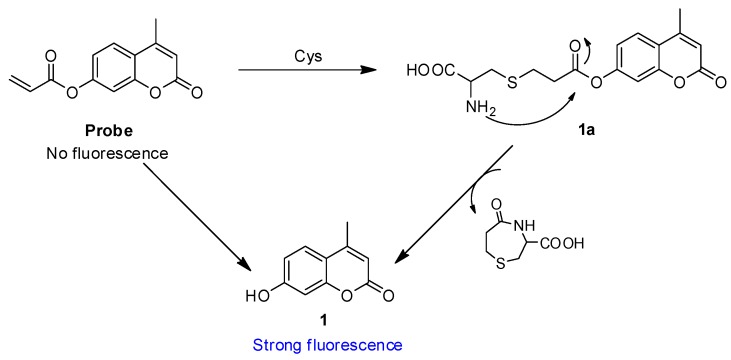
Proposed reaction mechanism of the probe with Cys.

The acrylate group has been applied as a thiols reaction site for detecting thiols [53,54]. Based on the reported conjugate addition/cyclization sensing mechanism, the mechanism of probe responding to Cys involved the next two steps: the conjugate addition of Cys to an unsaturated carbonyl moiety generated thioether (**1a**), followed by intramolecular cyclization that gave the desired compound **1**, which was responsible for the enhancement of the fluorescence intensity. To confirm the mechanism, the probe was incubated with Cys before being isolated by high performance liquid chromatography (see Figure S7 in the supporting information) and analyzed by mass spectra. In HPLC, a peak at 4.54 min corresponded to the compound **1** (coumarin) and a peak at 6.05 min corresponded to the probe. Furthermore, the probe detected compound **1** (coumarin) after being incubated with Cys for 3 h. In MS, a peak at 177.0 corresponded to the resulting compound **1** and a peak at 352.1 corresponded to the additional product compound **1a** (see Figure S8 in the supporting information). These data strongly support the mechanism in Scheme 2.

### 2.8. Application of the Probe

**Figure 7:**
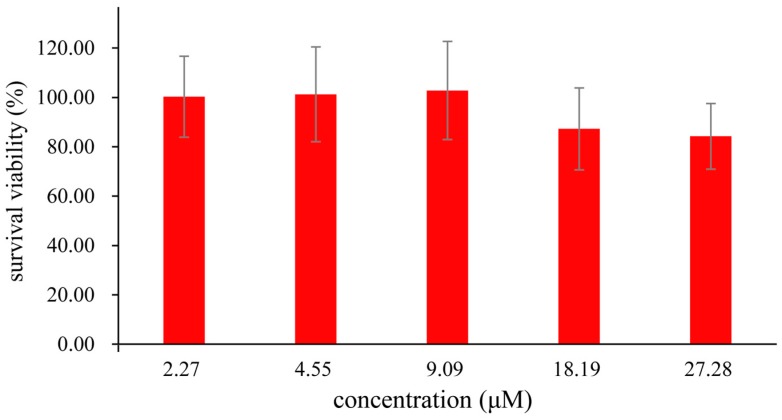
MTS assay of HepG2 cells incubated in the presence of the probe (0–27 μM) at 37 °C for 24 h.

**Figure 8:**
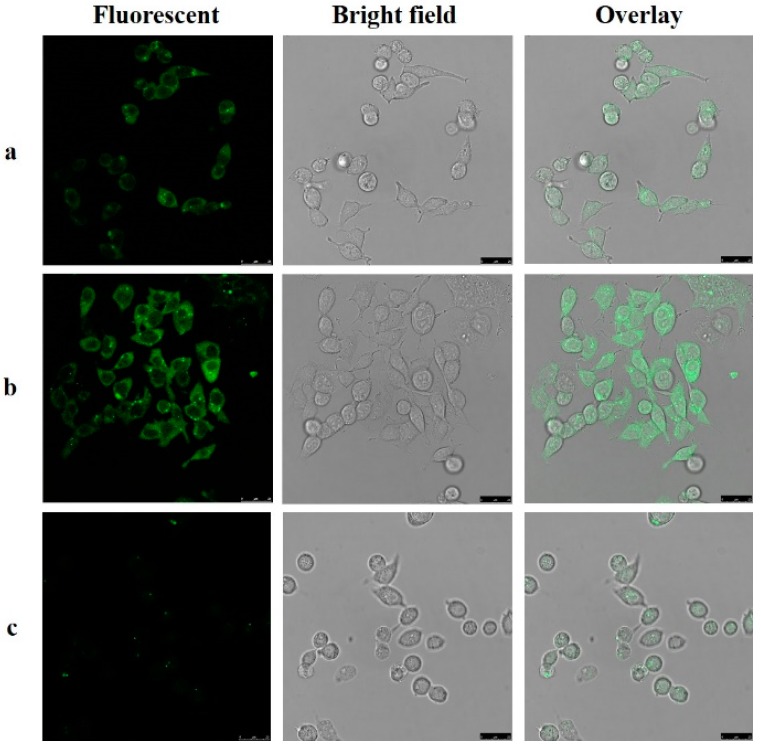
Fluorescent imaging of HepG2 cells: (Left) fluorescent image; (Middle) bright field image; and (Right) overlay image. λ_ex_ = 405 nm, λ_em_ = 420–480 nm. (**a**) Images of cells incubated with the probe (25 μM) for 2 h at 37 °C; (**b**) Images of cells preincubated with Cys (100 μM) for 1 h and then incubated with the probe (25 μM) for 2 h at 37 °C; (**c**) Images of cells preincubated with *N*-ethylmaleimide (NEM) (500 μM) for 1 h and then incubated with the probe (25 μM) for 2 h at 37 °C.

An MTS assay with a HepG2 cell line was applied to estimate the cytotoxicity of the probe. As shown in Figure 7, cellular viability was greater than 80% after 24 h in 27 μM (based on DMSO < 0.1%), which indicated that the probe had low toxicity to HepG2. To investigate the probe’s capability for cell imaging, HepG2 were incubated with the probe (25 μM) in PBS for 2 h at 37 °C and washed three times with PBS. As shown in Figure 8a, green fluorescence response could be observed inside cells under a confocal fluorescence microscope. This observation shows that the probe can detect the biothiols in the cells. Furthermore, in Figure 8b, when the cells were preincubated with Cys (100 μM) for 1 h and then incubated with the probe (25 μM), the fluorescence response was partly enhanced. In the control group, the cells were preincubated with 500 μM *N*-ethylmaleimide (NEM), a widely used thiol-blocking agent [55] for 1 h, then incubated with the probe (25 μM) for 2 h. As shown in Figure 8c, the fluorescence response was too slight to be detected, indicating the pertinence of Cys. The results obviously indicated that the probe could be used as a probe for both endogenously and exogenously produced Cys in living cells.

## 3. Materials and Methods

### 3.1. Materials and Instrumentation

All chemicals (reagent grade) used were purchased from Sino Pharm Chemical Reagent Co., Ltd. (Shanghai, China). Reaction progress was monitored using analytical thin layer chromatography (TLC) on pre-coated silica gel GF254 (Qingdao Haiyang Chemical Plant, Qingdao, China) plates, and spots were detected under UV light (254 nm). Melting point was measured on an XT-4 micromelting point instrument and uncorrected. IR (KBr-disc) spectra were recorded by a Bruker Tensor 27 spectrometer (Bruker, German). ^1^H-NMR and ^13^C-NMR spectra were measured on a BRUKER AVANCE III spectrometer at 25 °C and referenced to tetramethyl silane (TMS) (Bruker, German). Mass spectra were obtained on an MS Agilent 1100 Series LC/MSD Trap mass spectrometer (Agilent, Santa Clara, CA, USA). UV-vis spectra were recorded on an Agilent 8454 UV-vis spectrometer (Agilent). Fluorescence spectra measurements were recorded on an Agilent G9800A fluorescence spectrophotometer (Agilent). Fluorescence images were obtained on a Leica TCS-SP8 multiphoton; a confocal microscope and a 63× oil-immersion objective lens was used (Leica, Switzerland). High-resolution EI mass spectra were recorded on an Agilent 6460 triple quad LC-MS mass spectrometer (Agilent).

### 3.2. Synthesis of the Probe (4-Methyl-2-oxo-2H-chromen-7-yl Acrylate)

A solution of compound **1** (200 mg, 1.14 mmol) and triethylamine (0.12 mL, 0.86 mmol) in dried CH_2_Cl_2_ (10 mL) was added to acryloyl chloride (205.5 mg, 2.27 mmol) at 0 °C under nitrogen. After stirring for 12 h at room temperature, the reaction mixture was diluted with CH_2_Cl_2_. The CH_2_Cl_2_ solution was washed with a saturated aqueous Na_2_CO_3_ solution, as well as water, and then dried over anhydrous Na_2_SO_4_. The solvent was evaporated under reduced pressure. The crude product was purified by column chromatography (SiO_2_, ethyl acetate/hexane = 1/5, *v*/*v*) to give the probe as a pale white solid. Yield: 305 mg (80%). IR ν_max_/cm^−1^ (KBr) 1721.36, 1616.37 (C=O), 1388.53, 1164.33; m.p. 151.1–153.4 °C; ESI/MS *m*/*z*: 231.0 [M + H]^+^; ^1^H-NMR (400 MHz, CDCl_3_) δ 7.65 (d, *J* = 8.6 Hz, 1H, H_5_ coumarin), 7.20 (d, *J* = 2.2 Hz, 1H, H_8_ coumarin), 7.16 (dd, *J* = 8.6, 2.3 Hz, 1H, H_6_ coumarin), 6.68 (dd, *J* = 17.3, 1.1 Hz, 1H, H_3_ coumarin), 6.40–6.29 (m, 2H, H_3_ propionyl-), 6.11 (dd, *J* = 10.5, 1.1 Hz, 1H, H_2_ propionyl-), 2.47 (d, *J* = 1.2 Hz, 3H, -CH_3_). ^13^C-NMR (100 MHz, CDCl_3_) δ 163.8, 160.6, 154.2, 153.0, 152.0, 133.7, 127.3, 125.4, 118.1, 117.9, 114.6, 110.5, 18.7.

### 3.3. Absorption and Fluorescence Spectroscopy

The probe was dissolved in DMSO (1 mM) for a stock solution. The compounds such as cysteine (Cys), homocysteine (Hcy), glutathione (GSH), glycine (Gly), lysine (Lys), histidine (His), alanine (Ala), tyrosine (Try), cystine (Cys C), valine (Val), isoleucine (Ile), glutamic (Glu), phenylalanine (Phe), serine (Ser), threonine (Thr), aspartic (Asp), methionine (Met), proline (Pro), leucine (Leu), CN^−^, SCN^−^, HS^−^, and SO_4_^2−^ were all dissolved in purified water (10 mM). Tested solutions were prepared by adding 40 μL stock solution and a different analyte solution into the buffer (PBS:DMSO = 6:4, pH = 7.4) to a confirmed volume of 4 mL. The resulting solutions were mixed well and detected after 30 min at room temperature.

Fluorescent quantum yields were determined using Quinine sulphate (Ф_s_ = 0.54 in 0.1 M of H_2_SO_4_ solution) as a standard, according to a published method [56]. The fluorescent quantum yield was calculated based on the following equation:

Φ_x_ = Φ_s_ × (F_x_/F_s_) × (A_s_/A_x_) × (η_x_^2^/η_s_^2^) (2)

where Φ is the fluorescent quantum yield, Φ_s_ = 0.54 in 0.1 M of H_2_SO_4_ solution, F_x_ and F_s_ are the integrated fluorescence intensities of the sample and the standard, respectively, at the same excitation wavelength, A_x_ and A_s_ are the absorbance at the excitation wavelength, and η is the refractive index of the respective solvent.

### 3.4. Cell Culture for HepG2

HepG2 cells were provided by Chinese Academy of Sciences. HepG2 cells were cultured in minimum essential medium (MEM) blended with 10% fetal bovine serum (FBS) and 1% penicillin-streptomycin solution, (100×) at 37 °C under an atmosphere of 5% CO_2_. Cells were transplanted on confocal dishes and adhered for 24 h before use in an experiment.

### 3.5. Fluorescence Imaging of Cys in Living Cells

Experiments to evaluate the sense ability of the probe for biothiols were executed in culture medium (90% MEM, 10% FBS). The cells were incubated in the probe (25 μM) for 2 h at 37 °C and washed with 0.1 M PBS (0.6 mL × 3) before observation. To detect exogenously produced biothiols, cells were treated with Cys (100 μM) at 37 °C for 2 h and washed with 0.1 M PBS (0.6 mL × 3). Then, the cells were incubated in the probe (25 μM) for 2 h at 37 °C and washed with 0.1 M PBS (0.6 mL × 3) before observation. For the control experiment, cells were treated with 100 μM NEM for 1 h at 37 °C and washed with 0.1 M PBS (0.6 mL × 3). Then, the cells were incubated in the probe (25 μM) for 2 h at 37 °C and washed with 0.1 M PBS (0.6 mL × 3) before observation. For the positive experiment, cells were treated with coumarin chromophore (25 μM) for 2 h at 37 °C and washed with 0.1 M PBS (0.6 mL × 3) before observation. A fluorescence microscope and a 63× oil-immersion objective lens was used. The cells were excited with UV light below 405 nm, and emission was collected at 450 ± 40 nm.

## 4. Conclusions

The probe showed a stable, highly selective and sensitive fluorescence response towards Cys over GSH, Hcy, and other compounds. The main mechanism of the pertinence of Cys could be attributed to the nucleophilic addition and its lower steric hindrance. Confocal fluorescence microscopy imaging using HepG2 cells indicated that the probe can be applied for the detection of Cys in living cells.

## Figures and Tables

**Table 1:** Comparison of the probe for the detection of thiols.

Reference	Response Time	Stokes Shift	Detection Limit	Selectivity for Cys
Sensors and Actuators B, 2017 [46]	12 min	122 nm	17.1 nM	no
Sensors and Actuators B, 2016 [47]	3 h	110 nm	192 nM	no
Biosensors and Bioelectronics, 2017 [48]	20 min	85 nm	14 nM	yes
Biosensors and Bioelectronics, 2016 [49]	30 min	96 nm	0.874 μM	no
Biosensors and Bioelectronics, 2014 [50]	40 min	55 nm	0.657 μM	yes
ChemComm, 2013 [51]	2 h	97 nm	-	no
Molecules, 2016 [52]	2.5 h	174 nm	0.911 μM	no
This work	30 min	125 nm	47.7 nM	yes

## Supplementary Materials

The supplementary materials are available online.
