# Supplementary material for: A Fluorescent Coumarin-Based Probe for the Fast Detection of Cysteine with Live Cell Application
Source: Molecules. 2017 Sep 26;22(10):1618. doi: 10.3390/molecules22101618 (PMC6151380; doi:10.3390/molecules22101618)
Supplement: Supplementary file 1 [file molecules-22-01618-s001.pdf]

Supplementary data

A fluorescent coumarin-based probe for the fast detection of cysteine with live cell application

Rui-Feng Zeng[1†], Jin-Shuai Lan[2†], Xiao-Die Li[1], Hui-Fen Liang[1], Yan Liao[1], Ying-Jie Lu[1], Tong Zhang[1*], Yue Ding[2*]

1 School of Pharmacy, Shanghai University of Traditional Chinese Medicine, Shanghai 201203, China;

2 Experiment Center of Teaching & Learning, Shanghai University of Traditional Chinese Medicine, Shanghai 201203, China.

* Corresponding author. Tong Zhang; Yue Ding

* E-mail: zhangtongshutcm@hotmail.com (TZ); dingyue-2001@hotmail.com (YD)

† These authors contributed equally to this work.

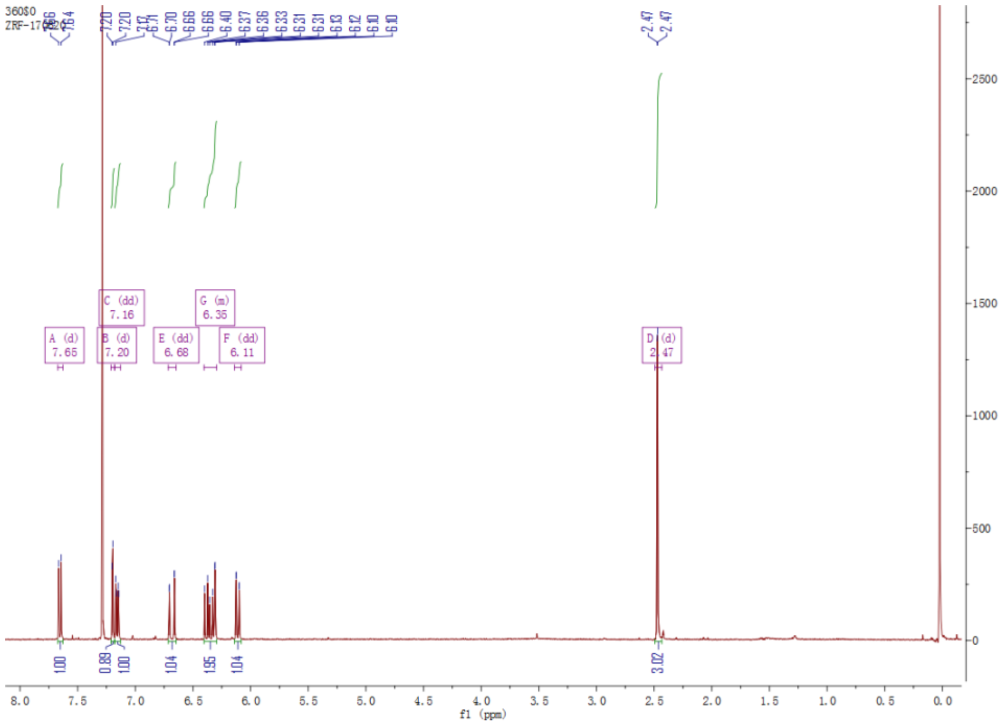

Fig S1. ^{1}H-NMR spectrum of the probe in $CDCl_3$.

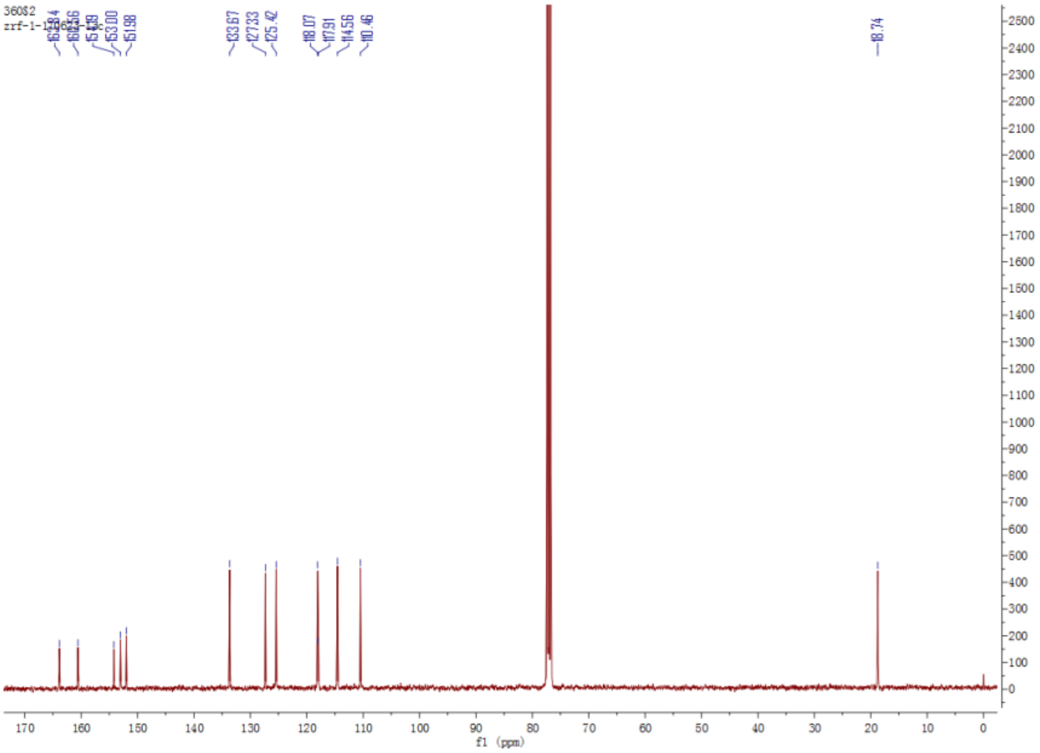

Fig S2. ^{13}C-NMR spectrum of compound the probe in $CDCl_3$.

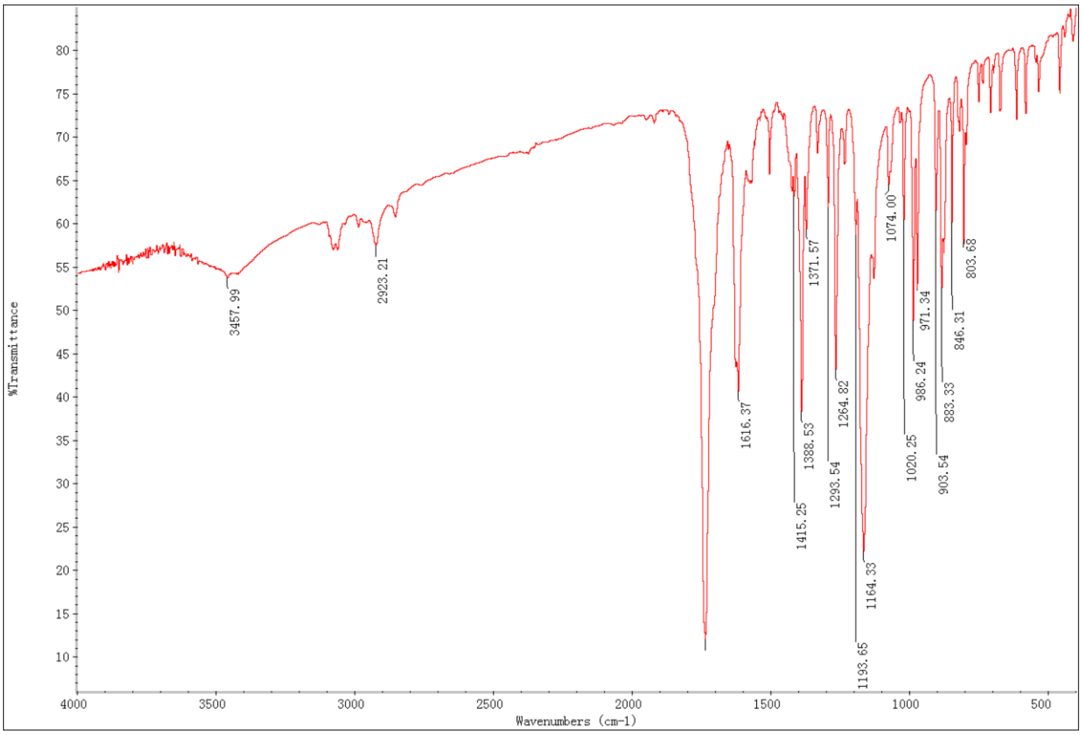

Fig S3. IR spectrum of compound the probe.

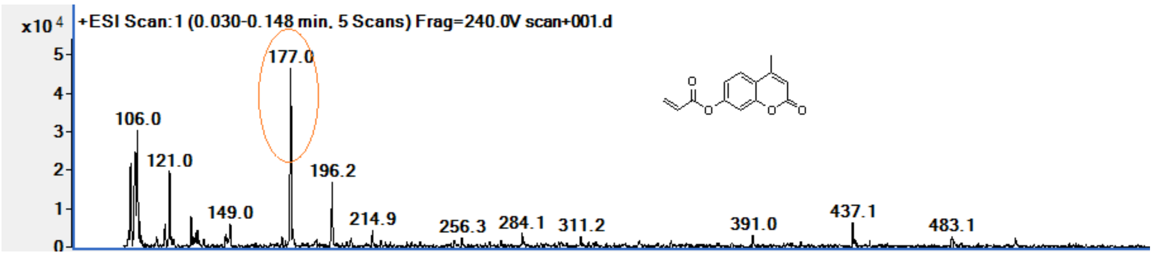

Fig S4. Mass spectrum of compound the probe.

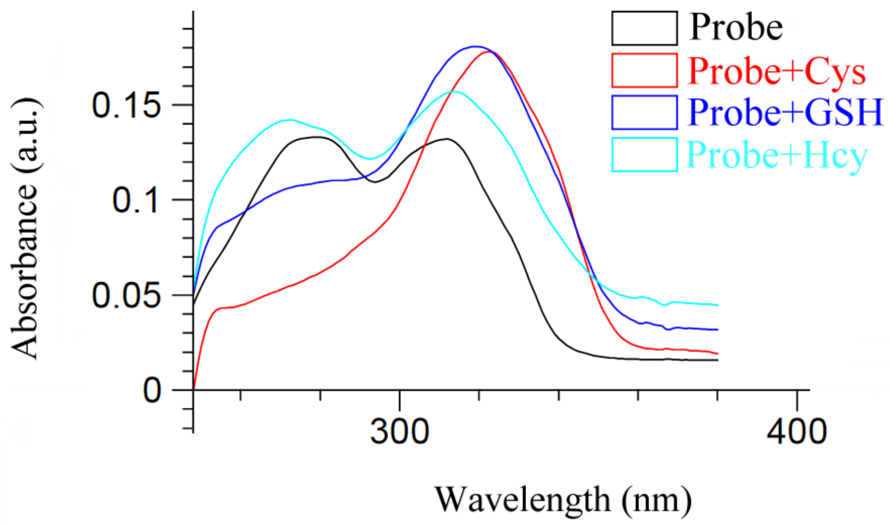

Fig S5. The absorption spectra of the probe and the probe added to Cys, GSH and Hcy in buffered solution (PBS:DMSO = 6: 4, PH = 7.4) at room temperature.

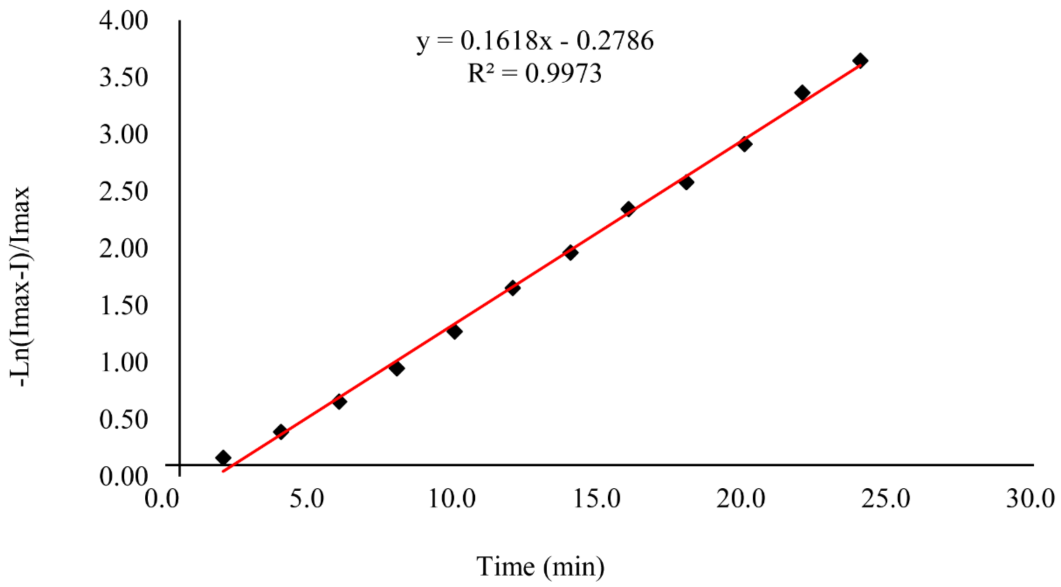

Fig S6. Pseudo-first-order rate figures of 10 μM probe in the presence of 50 μM Cys.

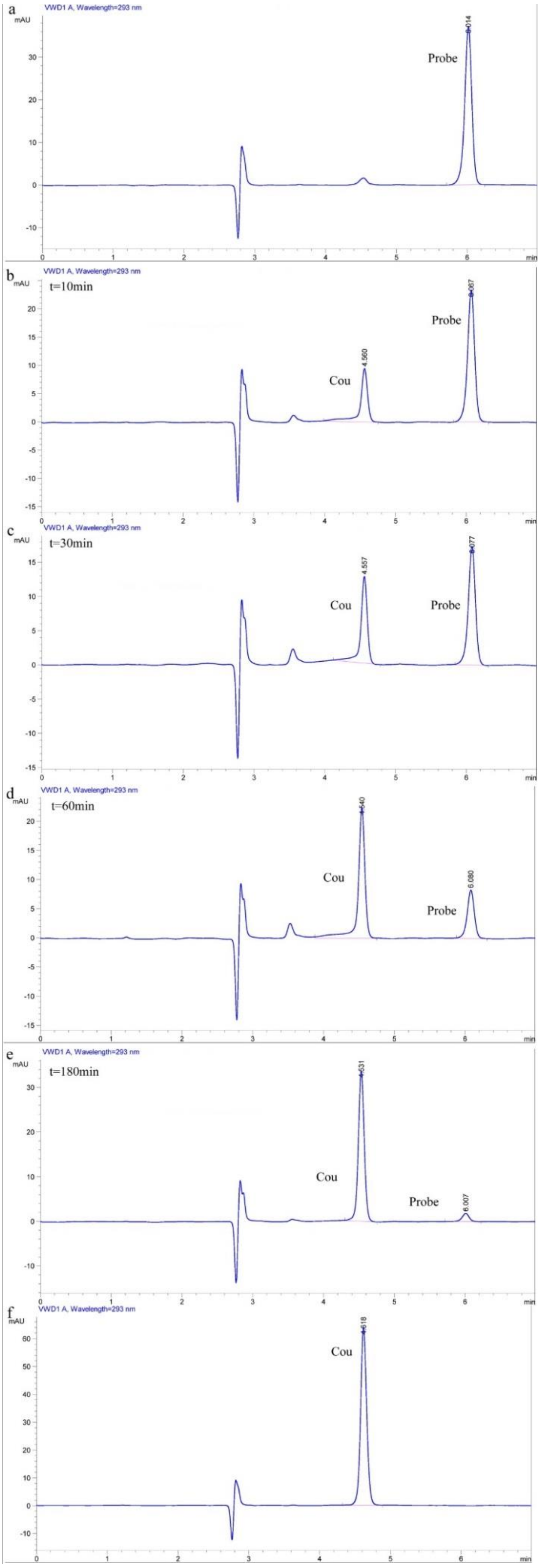
a
VWD1 A, Wavelength=293 nm
mAU
Probe
b
VWD1 A, Wavelength=293 nm
mAU
t=10min
Cou
4.560
Probe
c
VWD1 A, Wavelength=293 nm
mAU
t=30min
Cou
4.557
Probe
d
VWD1 A, Wavelength=293 nm
mAU
t=60min
Cou
Probe
6.080
e
VWD1 A, Wavelength=293 nm
mAU
t=180min
Cou
Probe
6.007
f
VWD1 A, Wavelength=293 nm
mAU
Cou

Figure S7. HPLC chromatogram in the reaction of probe (a):probe (50 μM) in PBS (pH 7.4) buffer; (b): probe (50 μM) reacted with Cys (10 equiv.) for 10min; (c): probe (50 μM) reacted with Cys (10 equiv.) for 30min; (d): probe (50 μM) reacted with Cys (10 equiv.) for 60min; (e): probe (50 μM) reacted with Cys (10 equiv.) for 160min; (f): Coumarin (50 μM) in PBS (pH 7.4) buffer.

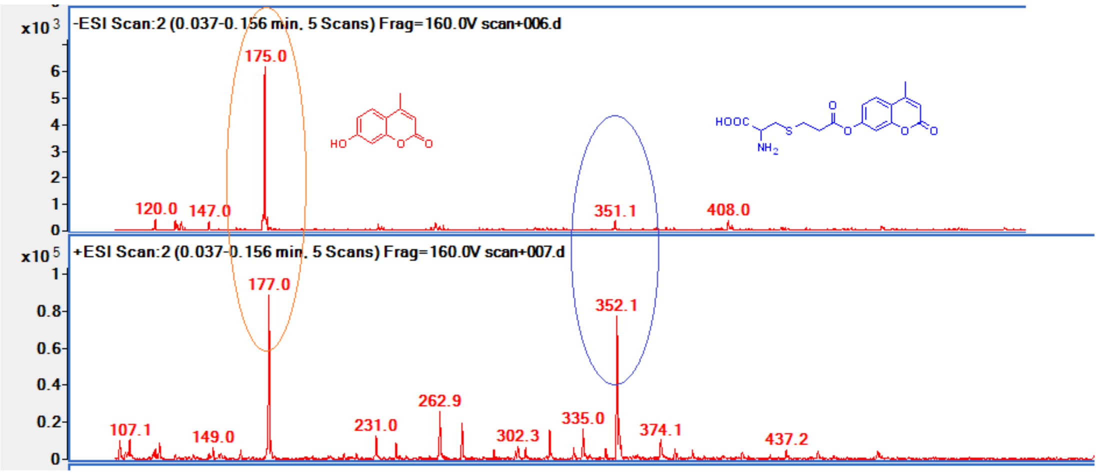

Figure S8. Mass spectrum of the crude product from the reaction of probe with Cys.
